# Postpartum depression childbirth related PTSD maternal bonding sexual functioning and partner support among mothers

**DOI:** 10.1038/s41598-026-41725-7

**Published:** 2026-02-27

**Authors:** Renáta Kovács-Berta, Lilla Sándor, Fanni Dudok, Norbert Pásztor, Edina Dombi

**Affiliations:** 1 Albert Szent-Györgyi Medical School, Doctoral School of Clinical Medicine, University of Szeged, Szeged, 6721 Hungary; 2 Juhász Gyula Faculty of Education, University of Szeged, Szeged, 6725 Hungary; 3 Department of Obstetrics and Gynaecology, University of Szeged, Szeged, 6725 Hungary

**Keywords:** Postpartum depression, Childbirth-related PTSD, Maternal-infant bonding, Sexual functioning, Partner support, Maternal self-efficacy, Depression, Anxiety, Risk factors

## Abstract

This study examined psychosocial factors associated with postpartum mental health, focusing on depression, childbirth-related PTSD (CB-PTSD), maternal-infant bonding, sexual functioning, and perceived partner support. Data from a large Hungarian sample (*N* = 675), assessed between 1 and 24 months postpartum indicated that 29.6% of mothers reported clinically significant depressive symptoms, 32.1% experienced sexual dysfunction, and 4.6% met diagnostic criteria for CB-PTSD, with substantial comorbidity observed between depression and PTSD. Bonding difficulties were consistently associated with higher depressive symptoms, lower perceived social support, and reduced maternal self-efficacy across all PBQ subscales, underscoring the central role of these factors in postpartum psychological adjustment. Sexual dysfunction was also associated with multiple domains of bonding difficulties, including impaired bonding, caregiving anxiety, and rejection-related distress, extending current understanding of postpartum relational dynamics. Regression analyses identified perceived partner care and relationship satisfaction as key correlates of maternal well-being, while emotional and instrumental forms of support were negatively associated with relationship satisfaction. Sociodemographic and obstetric factors, including emergency caesarean delivery and high-risk pregnancy, were further associated with increased psychological distress, highlighting their contextual relevance. These findings underscore the multidimensional nature of postpartum adjustment and suggest the relevance of integrated, trauma-informed, couple-focused approaches in perinatal mental health care.

## Introduction

Postpartum depression (PPD) is a major global mental health concern, affecting approximately 17–22% of women and significantly impairing maternal well-being and family functioning^1,2^. While its average prevalence within the first 6 months postpartum is estimated at around 13%^3^, a meta-analysis^4^ reported a global prevalence of 17.22%, with national rates ranging from 3% in Singapore to 38% in Chile^5^. Established risk factors include prior depression, antenatal anxiety, chronic stress, low social support, and socioeconomic inequality^4,5^. Prevalence rates tend to be higher in low-income (25.8%) and middle-income countries (20.8%) than in high-income countries (13–19%)^2,6^. During the COVID-19 pandemic, PPD rates increased globally, reaching 30.5% in high-income and 31.5% in low- and middle-income countries, compared with pre-pandemic estimates of 12–15%^7^. In Hungary, classified as a high-income country^8^, national studies estimate PPD prevalence between 7.1% and 10.8%, with key risk factors including poor living conditions, lack of emotional support, and antenatal depression^9,10^. Although screening programs in high-income countries promote early detection, barriers such as stigma, fear of judgment, and limited mental health literacy often deter women from seeking help^6,10–18^. Many women perceive depression symptoms as a normative aspect of the postpartum period, which may further delay treatment initiation^19^.

PPD is strongly associated with reduced maternal quality of life, relationship strain and disruptions in mother–infant bonding^20,21^. Maternal bonding, shaped by multiple psychosocial factors^22^, may be compromised by early separation, depressive symptoms, or traumatic birth experiences^23–26^. Childbirth-related post-traumatic stress disorder (CB-PTSD) frequently co-occurs with PPD (*r* = 0.63)^27,28^ and is associated with impaired bonding and relational functioning^29,30^. Low social support further exacerbates both depressive and post-traumatic symptoms^29,31^. The prevalence of postpartum PTSD is estimated to range between 0% and 7%, with most studies reporting rates of approximately 1–2%^32^.

According to attachment theory, individuals’ attachment orientation and their capacity to perceive and utilize partner support under stress play a central role in emotional regulation and relationship functioning^33^. In the perinatal context, perceived partner support is a key protective factor in postpartum adjustment, buffering stress and reducing the risk of depressive symptoms and bonding difficulties^29^. Supportive partners may also enhance maternal self-efficacy and responsiveness, thereby fostering healthier parent-infant interaction^34^, whereas low partner involvement predicts poorer sensitivity and higher psychological distress^35^. Secure maternal-infant bonding is more likely when mothers feel competent and supported, as high maternal self-efficacy may protect against bonding disruptions and mitigate intergenerational stress transmission^36^. Partner support has been shown to consistently predict stronger bonding and more favorable socio-emotional outcomes in children^37,38^. While maternal stress and depressive symptoms are associated with poorer bonding quality^39^, greater partner support is linked to reduced maternal distress^40^. In contrast, miscarriage history alone does not appear to predict bonding difficulties^41^.

Sexual dysfunction is represents another frequently reported challenge in the postpartum period. Chivers et al. found that approwimately 40% of women with PPD experience difficulties related to sexual desire, arousal, or orgasm, which may persist even after remission of depressive symptoms^21^. Other studies have identified high EPDS scores, perineal pain, and low relationship satisfaction as significant predictors of postpartum sexual difficulties^42^. Chang et al. suggested a bidirectional association, noting that sexual dysfunction may precede depressive symptoms, while higher income and greater sexual satisfaction may serve as protective factors^43^. A recent review by Kelley emphasized that relationship quality and subjective sexual experience are stronger predictors of postpartum sexual well-being than depressive symptoms alone^44^, underscoring the relational dimension of postpartum adjustment.

Higher levels of social support are consistently associated with lower PPD prevalence and greater maternal well-being^2,45^. Conversely, insufficient support increases stress and psychological vulnerability^46,47^, while prenatal support predicts better bonding and fewer depressive symptoms^3,46,48,49^. Liu et al. further demonstrated that social support and family structure may buffer against both PPD and postpartum PTSD^4^. Relationship quality also plays a critical role in maternal adjustment, as supportive relationships are associated with fewer depressive symptoms and better psychological outcomes, whereas controlling or critical partner behaviors increase vulnerability. Higher partner care, as measured by the Intimate Bond Measure, has been linked to improved maternal mental health and relationship satisfaction in the postpartum period^50^.

The present study examines interrelations between postpartum depression, childbirth-related PTSD (CB-PTSD), maternal-infant bonding, sexual functioning, maternal self-efficacy and perceived partner support. Despite growing recognition of these factors, few studies have investigated them within a unified analytical framework, particularly in Central and Eastern Europe, including Hungary. This gap limits understanding of how sociocultural and relational factors jointly shape postpartum adjustment. Accordingly, the study aimed to investigate how these psychological and interpersonal dimensions are associated with maternal bonding and sexual functioning, with particular attention to the roles of maternal self-efficacy and partner support in postpartum mental health. Based on prior literature, we hypothesized that (1) lower levels of perceived partner support and maternal self-efficacy are associated with greater bonding difficulties and more severe depressive symptoms, and (2) CB-PTSD symptoms are significantly associated with sexual dysfunction and impairments in maternal-infant bonding.

## Methods

The study was approved by the Ethics Committee of the University of Szeged (reference number: 133/2021-SZTE RKEB) and conducted in accordance with the principles of the Declaration of Helsinki.

### Sample

Participants were recruited online using convenience sampling. The study sample consisted exclusively of postpartum women. Aged 18 years or older, fluent in Hungarian, and who had given birth within the previous 2 years. Of the 685 respondents who completed the full questionnaire, 10 were excluded because their childbirth had occurred more than 2 years earlier, resulting in a final sample of 675 participants.

### Measures

Sociodemographic and obstetric data included maternal age, education level, marital status, relationship duration, parity, number of children, pregnancy planning, mode of delivery, obstetric complications, and pregnancy risk status, the latter as documented by the attending physician.

*Edinburgh Postnatal Depression Scale (EPDS)* A 10-item screening tool assessing depressive and anxiety symptoms postpartum, scored on a 0–3 scale. In Hungary, scores of 8–12 indicate mild and scores ≥ 13 indicate major depression^51–53^.

*Beck Depression Inventory (BDI)* A 21-item self-report measure of depressive symptoms rated on a 0–3 scale. Scores of 10–18 indicate mild, 19–25 moderate, and ≥ 26 severe depression^54,55^.

*City Birth Trauma Scale (City BiTS)* Childbirth-related PTSD symptoms were assessed using the 29-item City BiTS, developed to measure PTSD according to DSM-5 criteria. The scale assesses trauma exposure (Criterion A), symptom frequency across the four DSM-5 clusters (re-experiencing, avoidance, negative mood and cognitions, and hyperarousal; Criteria B-E), as well as symptom duration and associated distress (Criteria F-G). Items are rated on a 0–3 Likert scale (0 = not at all, 3 = five or more times in the past week), with higher scores indicating greater symptom severity. Two items identify the dissociative subtype. A total symptom severity score (range 0–60) and two subscale scores can be computed: Birth-Related Symptoms (items 3–12) and General Symptoms (items 13–22). The scale allows both dimensional assessment and categorical diagnosis and demonstrates excellent internal consistency (Cronbach’s α = 0.92)^32^.

*Intimate Bond Measure (IBM-HU)* Assesses perceived partner care and control using 24 items rated on a 0–3 Likert scale. The original IBM was developed by Wilhelm and Parker^56^. The Hungarian adaptation demonstrates strong reliability (Care α = 0.94; Control α = 0.91)^57^.

*Relationship Assessment Scale (RAS-H)* Evaluates subjective relationship satisfaction using seven items rated on a 1–5 Likert scale. The original scale was developed by Hendrick et al.^58^, and the Hungarian version demonstrates high internal reliability (α = 0.843–0.897)^59^.

*General Self-Efficacy Scale (Hungarian version)* Consists of 10 items assessing perceived competence in stressful situations, rated on a 1–4 scale. Higher scores indicate greater self-efficacy, with good internal consistency (α = 0.88)^60^.

*Satisfaction with Life Scale (SWLS-H)* A five-item measure of general life satisfaction rated on a 1–7 scale, with higher scores reflecting greater satisfaction (α = 0.84)^61^.

*WHO-5 Well-Being Index (Hungarian version)* Evaluates subjective well-being using five items rated on a 0–3 scale, yielding total scores between 0 and 15, with higher scores indicating better well-being. The original WHO-5 was developed by the World Health Organization^62^ and the Hungarian validation demonstrates good internal consistency (α = 0.85)^63^.

*Female Sexual Function Index (FSFI-H)* Assesses female sexual functioning across six domains (desire, arousal, lubrication, orgasm, pain, satisfaction) using 19 items. Total scores range from 2 to 36, with lower scores indicating greater dysfunction. A cut-off score of 26.55 indicates clinically significant dysfunction^64^. The Hungarian version was validated by Hock et al. and shows excellent internal consistency (α = 0.963)^65^.

*Postpartum Bonding Questionnaire (PBQ)* A 25-item scale measuring mother–infant bonding across four domains: impaired bonding, rejection and anger, caregiving anxiety, and risk of abuse. Items are rated from 0 (always) to 5 (never), with higher scores indicating greater bonding difficulties (α = 0.83)^66^.

*MOS Social Support Survey (MOS-SSS-H)* Includes 20 items assessing emotional and informational support, tangible support, and positive social interaction on a 1–5 Likert scale. The original scale was developed by Sherbourne and Stewart^67^, and the Hungarian adaptation demonstrates excellent psychometric properties (α = 0.89 to 0.95)^68^.

### Translation and adaptation

Both the City BiTS and PBQ were translated and culturally adapted for use in Hungarian postpartum samples. The Hungarian adaptation followed Sousa and Rojjanasrirat’s seven-step translation methodology, including forward translation, expert review, and back-translation with cultural and linguistic adjustments^69^. Pretesting with 12 mothers confirmed clarity and comprehensibility. Detailed psychometric results of the City BiTS Hungarian version are reported in separate manuscript currently under editorial review. The PBQ was administered up to two years postpartum, consistent with emerging evidence that bonding processes extend beyond the first year^70^.

### Procedure

Women aged ≥ 18 years, fluent in Hungarian, and within two years postpartum were invited to participate. Due to COVID-19 pandemic-related restrictions, data collection occurred online between November 2021 and November 2022. The survey link was distributed via the Hungarian health-visitor network and through social media groups focused on maternal and postpartum topics, ensuring recruitment from the target postpartum female population. Participation was voluntary and uncompensated. Before beginning the survey, participants received detailed study information, including the possibility of encountering sensitive content, and information on available psychological support resources. Electronic informed consent was obtained, and participants were reminded that withdrawal was possible at any time. Completion required approximately 20–25 min, and responses were anonymous to ensure confidentiality. During data collection (2021–2022), Hungary was classified by the World Bank as a high-income country^8^.

### Statistical analysis

All analyses were conducted using IBM SPSS Statistics version 22.0, with the significance threshold set at *p* < 0.05 (two-tailed). Descriptive statistics (means, standard deviations, and frequencies) were used to summarize sample characteristics and questionnaire scores. Pearson’s correlation coefficients were calculated to examine associations among continuous psychological, relational, and social support variables variables.

Independent-samples t tests were performed to compare groups by parity, time since childbirth, and pregnancy risk status. Multivariate analyses of variance (MANOVA) were employed to assess the combined effects of depressive symptoms, bonding difficulties, and sexual functioning on caregiving perceptions and quality of life indicators. MANOVA was selected to minimize Type I error and to account for intercorrelated dependent variables.

Multiple linear regression analyses were conducted to examinde predictors of postpartum bonding (PBQ), perceived social support (MOS-SSS), and relationship satisfaction (RAS-H) Predictor selection was theory-driven, based on prior findings highlighting the role of self-efficacy, depressive and PTSD symptoms, and partner support in postpartum adjustment^29,33,40^. Covariates included maternal age, parity, relationship duration, and time since childbirth. The models incorporated psychological (EPDS, BDI, City BiTS, self-efficacy, FSFI-H, WHO-5, SWLS-H, IBM Care and Control), demographic (age, relationship duration, education, marital status) and obstetric variables (pregnancy risk, pregnancy complications, and mode of delivery) to esimate associations between psychological factors and postpartum outcomes while accounting for demographic and medical influences.

Subgroup analyses were conducted for clinically and theoretically relevant contextual factors, including relationship duration, mode of delivery, pregnancy risk status, and specific obstetric complications. Relationship duration was recoded into three categories (≤ 3 years, 4–10 years, > 10 years) to represent early, mid-, and long-term partnerships^33^. Comparison groups (e.g., high- vs. low-risk pregnancy) were analyzed to ensure interpretive balance. Participants were further classified according to clinically relevant cut-off criteria (e.g., presence vs. absence of clinically significant depressive symptoms of CB-PTSD).

Assumptions of normality, linearity, and homogeneity of variance were met. Results are presented across four main sections: (1) prevalence of psychological and relational indicators, (2) bivariate and adjusted associations, (3) subgroup analyses, and (4) multivariate regression models. The presentation emphasizes statistically robust and clinically relevant findings.

## Results

### Overview

This section presents descriptive and prevalence data, followed by bivariate and adjusted associations. Subgroup analyses and multivariable regression models are subsequently reported to identify predictors of maternal bonding, perceived social support, and relationship satisfaction.

### Sample characteristics and contextual factors

The final sample consisted of 675 postpartum women (M_age = 32.02 years, SD = 4.74). Most participants lived in urban areas (60.4%), held a college or university degree (53.6%), and were married (87.1%). The majority were multiparous (89.2%), and 88.7% reported planned pregnancies. Regarding birth characteristics, 55.3% experienced spontaneous vaginal delivery, while 42.1% gave birth via caesarean Sect.  (31.3% planned, 10.8% emergency). Pregnancy complications were reported by 36%, and 44.3% required additional specialist consultations. The mean gestational age was 39 weeks (SD = 1.84), and the average birth weight was 3401.19 g (SD = 553.31) (Table 1).

The classification of „high-risk pregnancy” was determined by the attending obstetrician, based on clinical judgment rather than solely on the presence of obstetric complications. Consequently, some mothers with health contidions (e.g., gestational diabetes or infections) were not categorized as high-risk when no additional monitoring or intervention was required. Overall, the sample primarily comprised highly educated, urban-dwelling women in stable relationships, most of whom experienced planned pregnancies.

**Table 1:** Demographic and obstetric characteristics of the sample (*N* = 675).

Variable	Category/description	*N* (%)/M (SD)
Maternal age (years)	Mean (SD), range	32.02 (4.74), 19–48
Place of residence	Capital city	76 (11.3)
	City	408 (60.4)
	Village	179 (26.5)
	Municipality/farm	12 (1.7)
Education level	Below graduation	29 (4.3)
	Upper secondary	271 (40.1)
	University/college	362 (53.6)
	Postgraduate/doctorate	13 (1.9)
Marital status	Married	588 (87.1)
	Cohabiting	80 (11.9)
	Divorced/single	7 (1.0)
Relationship duration (years)	< 1	1 (0.1)
	1–3	76 (11.3)
	4–6	175 (25.9)
	7–10	206 (30.5)
	11–15	148 (21.9)
	> 15	65 (9.6)
Parity	Primiparous	73 (10.8)
	Multiparous	602 (89.2)
Number of children	Mean (SD)	1.56 (0.98)
Planned pregnancy	Yes	599 (88.7)
	No	76 (11.3)
Mode of conception	Natural	648 (96)
	Assisted (ART)	27 (4)
Pregnancy type	Low-risk	424 (62.8)
	High-risk	251 (37.2)
Gestational age (weeks)	Mean (SD), range	39.01 (1.84), 23–42
Apgar score (5 min)	Mean (SD)	9.16 (1.57)
Birth weight (g)	Mean (SD), range	3401.19 (553.31), 650–5150
Delivery mode	Vaginal	373 (55.3)
	Forceps/vacuum	17 (2.7)
	Elective caesarean	211 (31.3)
	Emergency caesarean	73 (10.8)
Need for increased medical care	Yes	299 (44.3)
Pregnancy complication	None	432 (64)
	Threatened preterm birth	21 (3.1)
	Fetal growth restriction	11 (1.6)
	Hypertension	30 (4.4)
	Gestational diabetes	69 (10.2)
	Recurrent miscarriages	28 (4.1)
	Infection (viral/bacterial)	28 (4.1)
	Other complications	42 (6.2)

Continuous variables are presented as mean (M) and standard deviations (SD), unless otherwise indicated; categorical variables are presented as N (%). ART = Assisted Reproductive Technology. Apgar score refers to the standardized assessment of newborn health 5 min after delivery. High-risk pregnancy refers to pregnancies requiring intensified medical monitoring due to obstetric or medical complications (e.g. hypertension, diabetes, infection or fetal growth restriction). Need for increased medical care includes more frequent specialist consultations, laboratory investigations, or inpatient observation.

### Prevalence of postpartum psychological and relational difficulties

Descriptive analyses indicated that 26.4% of participants reported mild depressive symptoms, while 29.6% scored above the threshold for major depression on the EPDS. Based on the BDI, 30.4% reported mild, 13.2% moderate, and 8.9% severe depressive symptoms.

The mean CB-PTSD score was 11.60 (SD = 10.42), and 4.6% met DSM-5 criteria for childbirth-related PTSD. Severe bonding disturbances were observed in 1.6% of the sample, whereas clinically significant sexual dysfunction was present in 32.1% of participants.

These prevalence rates were higher than those reported in several pre-pandemic studies (Table 2).

**Table 2:** Descriptive statistics of psychological and relational measures in the postpartum sample (*N* = 675).

Measure	Mean	SD	95% CI (lower–upper)
BDI	12.09	8.759	11.43–12.81
EPDS	9.38	6.147	8.94–9.88
City BiTS	11.598	10.42	10.86–12.44
IBM care	27.21	8.397	26.65-27.826
IBM control	8.34	7.187	7.82–8.88
RAS-H	28.767	5.921	28.33–29.22
Self-efficacy	31.232	5.424	30.85–31.64
SWLS-H	26.63	5.908	26.15–27.08
WHO-5	9.62	3.693	9.34–9.9
FSFI-H	21.757	11.11	20.82–22.54
PBQ total	7.481	9.403	6.81–8.21
PBQ-1 impaired bonding	4.155	5.049	3.77–4.55
PBQ-2 rejection and anger	1.413	2.649	1.22–1.62
PBQ-3 anxiety about care	1.843	2.328	1.67–2.01
PBQ-4 risk of abuse	0.069	0.504	0.035–0.11
MOS-SSS-H total	82.124	6.011	80.796–83.305
MOS-SSS-H size of the social network	5.07	3.985	4.77–5.38
MOS-SSS-H-1 emotional/informational support	34.578	7.48	33.942–35.132
MOS-SSS-H-2 positive social interaction support	30.689	5.95	30.202–31.146
MOS-SSS-H-3 Instrumental support	16.857	3.905	16.548–17.15

Mean values and standard deviations (SD) are presented for each scale and subscale. 95% confidence intervals (95% CI) indicate the precision of the mean estimates. Higher scores reflect greater symptom severity or higher levels of the measured construct, depending on the instrument. Interpretation of clinically relevant cut-off scores is provided in the Methods section. *BDI* Beck Depression Inventory, *EPDS* Edinburgh Postnatal Depression Scale, *City BiTS* City Birth Trauma Scale, *IBM* Intimate Bond Measure, *RAS-H* Relationship Assessment Scale, *SWLS-H* Satisfaction With Life Scale, *WHO-5* WHO-5 Well-Being Index, *FSFI-H* Female Sexual Function Index, *PBQ* Postpartum Bonding Questionnaire, *MOS-SSS-H* Medical Outcomes Study Social Support Survey.

### Multivariate associations between depression, bonding, and sexual dysfunction

To examine the combined effects of depressive symptoms, bonding difficulties, and sexual functioning on relational and well-being indicators, a series of multivariate analyses of variance (MANOVAs) were conducted.

Bonding difficulties were negatively associated with perceived partner care (F(2,664) = 3.362, *p* = 0.035), indicating that greater bonding impairment was related to lower perceived partner support. Both depressive symptoms and sexual dysfunction were associated with reduced partner care (BDI: F(3,667) = 3.83, *p* = 0.01; FSFI: F(1,667) = 48.206, *p* < 0.001) and increased partner control (F(1,667) = 13.094, *p* < 0.001). Across models, bonding difficulties and sexual dysfunction were also negatively associated with self-efficacy (F(2,669) = 11.851, *p* < 0.001), life satisfaction (F(2,666) = 11.294, *p* < 0.001), and well-being (F(2,664) = 15.386, *p* < 0.001).

When focusing on bonding outcomes, depressive symptoms (EPDS) were positively associated with impaired bonding (F(2,669) = 6.37, *p* = 0.002) and caregiving anxiety (F(2,669) = 3.158, *p* = 0.043). Sexual dysfunction (FSFI) showed similar patterns, being positively associated with impaired bonding (F(1,669) = 29.418, *p* < 0.001), caregiving anxiety (F(1,669) = 21.81, *p* < 0.001) rejection and anger (F(1,669) = 21.054, *p* < 0.001) and increased risk of abuse (F(1,669) = 4.908, *p* = 0.027).

Taken together, the MANOVA results indicate that depressive symptoms and sexual dysfunction were consistently related to poorer bonding, lower relational quality, and diminished psychological well-being.

### Bivariate correlations among psychological, relational, and support variables

Pearson correlation coefficients were calculated to examine associations between psychological, relational, and social support measures (Table 3). The strongest associations were observed between perceived partner care and relationship satisfaction (*r* = 0.851), followed by the association between PTSD and depressive symptoms (*r* = 0.665).

Depressive symptoms (EPDS, BDI) were positively correlated with bonding difficulties across PBQ subscales (*r* = 0.239–0.282), and negatively correlated with life satisfaction (*r* = − 0.29 to − 0.238), total social support (*r* = − 0.238 to − 0.275), and instrumental support (*r* = − 0.278 to − 0.341). PTSD symptoms (City BiTS) were negatively associated with self-efficacy, life satisfaction, and perceived social support (*r* = − 0.235 to − 0.321, all *p* < 0.001).

Maternal self-efficacy was positively correlated with life satisfaction (*r* = 0.468), well-being (*r* = 0.566), sexual satisfaction (*r* = 0.343), and total social support (*r* = 0.350), while showing negative associations with bonding difficulties (*r* = − 0.417 to − 0.401; all *p* < 0.001).

Within the relationship domain, partner control (IBM-Control) was negatively correlated with relationship satisfaction (*r* = − 0.643), life satisfaction (*r* = − 0.392), and social support (*r* = − 0.418 to − 0.578). In contrast, partner care (IBM-Care) was positively correlated with relationship satisfaction (*r* = 0.851), life satisfaction (*r* = 0.583), and well-being (*r* = 0.414), and negatively correlated with bonding difficulties (*r* = − 0.272).

Sexual satisfaction, arousal, and orgasm showed moderate positive correlations with perceived partner care (*r* = 0.254–0.378, *p* < 0.001). Overall, depressive and PTSD symptoms were associated with greater bonding difficulties and lower psychosocial well-being, while maternal self-efficacy and partner care emerged as the most consistent protective factors.

**Table 3:** Pearson correlation coefficients between key psychological, relational, and social support variables (*N* = 675).

Measure	EPDS	City BiTS	IBM Care	RAS-H	SWLS-H	WHO-5	FSFI-H total	PBQ total	PBQ-1	PBQ-2	PBQ-3	PBQ-4	MOS-1	MOS-2	MOS-3	MOS total
BDI	0.782	0.665														
EPDS		0.624														
IBM Care	− 0.125			0.851	0.583	0.414	0.282		− 0.272				0.415	0.565	0.431	0.509
IBM Control				− 0.631	− 0.403	− 0.254							− 0.276	− 0.383	− 0.291	− 0.342
RAS-H					0.64	0.377	0.25		− 0.267				0.377	0.55	0.381	0.474
Self efficacy					0.339	0.449		− 0.345	− 0.32	− 0.299	− 0.332		0.299	0.321	0.265	0.323
SWLS-H						0.488		− 0.304	− 0.34				0.473	0.56	0.438	0.536
WHO-5							0.34	− 0.459	− 0.47	− 0.379	− 0.371		0.489	0.531	0.421	0.528
PBQ Total									0.956	0.908	0.832	0.461	− 0.279	− 0.294		− 0.295
PBQ 1.										0.812	0.689	0.372	− 0.297	− 0.317		− 0.317
PBQ 2.											0.685	0.378				
PBQ 3.												0.407				
MOS 1.														0.831	0.698	0.946
MOS 2.							0.264								0.756	0.944
MOS 3.																0.851

Displayed values are Pearson correlation coefficients (r). For clarity, only statistically significant correlations (*p* < 0.05) with an absolute of *r* ≥ 0.25 are shown; empty cells indicate correlations that did not meet these criteria. All tests were two-tailed. *BDI* Beck Depression Inventory, *EPDS* Edinburgh Postnatal Depression Scale, *City BiTS* City Birth Trauma Scale, *IBM Care* Intimate Bond Measure-Care dimension, *IBM Control* Intimate Bond Measure-Control dimension, *RAS-H* Relationship Assessment Scale (Hungarian version), *Self-efficacy* self-Efficacy Scale, *SWLS-H* Satisfaction With Life Scale (Hungarian version), *WHO-5* WHO-5 Well-Being Index, *FSFI-H* Female Sexual Function Index (Hungarian version), *PBQ Total* Postpartum Bonding Questionnaire-total score, *PBQ-1* impaired bonding, *PBQ-2* rejection and anger, *PBQ-3* anxiety about care, *PBQ-4* risk of abuse, *MOS Total* Medical Outcomes Study Social Support Survey-total score, *MOS-1* emotional/informational support, *MOS-2* positive social interaction, *MOS-3* instrumental support.

### Adjusted associations controlling for obstetrics and demographic factors

Partial correlation analyses were conducted to assess whether the bivariate associations remained significant after controlling for potential confounders. Obstetric (mode of delivery, pregnancy risk status, need for intensified medical care), demographic (maternal age, parity, time since childbirth), and psychological variables (EPDS, BDI, RAS-H) was entered as a control variables.

After adjustment, the association between PTSD symptoms and maternal bonding was no longer significant (*r* = 0.06, *p* = 0.105), whereas depressive symptoms measured by the EPDS remained significantly associated with greater bonding difficulties (*r* = 0.13, *p* = 0.001). The general depression measure (BDI) showed no independent association (*r* = − 0.07, *p* = 0.056). Higher perceived social support (*r* = − 0.29, *p* < 0.001), greater relationship satisfaction (*r* = − 0.22, *p* < 0.001), and better sexual functioning (*r* = − 0.15, *p* < 0.001) were indipendently associated with fewer bonding difficulties.

### Subgroup differences by relationship duration and perinatal risk factors

To examine differences in psychosocial indicators across relationship duration and perinatal risk groups, one-way analyses of variance and independent-samples t tests were conducted.

Mothers with high-risk pregnancies (*N* = 251) reported significantly higher BDI scores than those with low-risk pregnancies (*N* = 424; t(673) = − 2.30, *p* = 0.022; M = 13.10, SD = 9.36 vs. M = 11.50, SD = 8.34). No other psychological or relational variables differed significantly between pregnancy risk groups.

Across relationship duration categories, significant differences emerged in several psychosocial and sexual functioning domains. Post hoc Bonferroni-corrected tests indicated that women in mid-length relationships (4–10 years, *N* = 381) reported lower self-efficacy (F(2,672) = 4.19, *p* = 0.016; Bonfferoni *p* = 0.014) compared to those in long-term relationships (> 10 years, *N* = 213). The mid-length group also reported lower sexual arousal (F(2,672) = 4.38, *p* = 0.013; Bonfferoni *p* = 0.037), lubrication (F(2,672) = 4.59, *p* = 0.010; Bonfferoni *p* = 0.029), orgasm (F(2,672) = 4.39, *p* = 0.013; Bonfferoni *p* = 0.042), sexual satisfaction (F(2,672) = 4.95, *p* = 0.007; Bonfferoni *p* = 0.028), and total sexual functioning on the FSFI (F(2,672) = 4.49, *p* = 0.012; Bonfferoni *p* = 0.036) compared to the long-term relationship group. Conversely, the mid-length relationship group exhibited higher PTSD symptom scores (F(2,672) = 3.61, *p* = 0.028; Bonfferoni *p* = 0.023).

### Mode of delivery

Distinct association patterns emerged across delivery modes. Among women who had vaginal deliveries (*N* = 373), relationship satisfaction was positively correlated with perceived partner care (*r* = 0.847), self-efficacy, well-being, sexual functioning, and social support (*r* = 0.202–0.629), and negatively correlated with partner control and bonding difficulties (*r* = − 0.288 to − 0.600).

In the planned caesarean section group (*N* = 211), partner control (IBM-Control) was negatively correlated with relationship satisfaction and social support, whereas self-efficacy was negatively associated with bonding difficulties and positively associated with social support (*r* = − 0.361 to 0.305).

Among women who experienced emergency caesarean section (*N* = 73), depressive symptoms were positively correlated with caregiving anxiety (PBQ subscale 3; *r* = 0.262). Bonding disturbances in this group were negatively associated with perceived partner care, relationship satisfaction, self-efficacy, life satisfaction, and well-being (*r* = − 0.250 to − 0.592), and positively associated with partner control (*r* = 0.319) (Table 4).

**Table 4:** Associations between key postpartum adjustment indicators within delivery mode subgroups.

Subgroup	Key Variable Associations
Vaginal birth (*N* = 373)	**RAS-H** with IBM Care (*r* = 0.847), SWLS-H (*r* = 0.629), FSFI-H Total (*r* = 0.259), self-efficacy (*r* = 0.202), WHO-5 (*r* = 0.391), and MOS-SSS Total (*r* = 0.546; MOS-1 to MOS-3: *r* = 0.454–0.613), negative associations with IBM Control (*r* = − 0.600); PBQ Total (*r* = − 0.242; PBQ-1: *r* = − 0.288)
Planned caesarean (*N* = 211)	Negative associations between **IBM Control** and RAS-H (*r* = − 0.638), and MOS-SSS Total (*r* = − 0.275, MOS-2: (*r* = − 0.370).); **Self-efficacy** was negatively associated with PBQ-1 to PBQ-3 (*r* = − 0.334 to − 0.361), and positively associated with MOS-SSS Total and subscales (*r* = 0.268–0.305)
Emergency caesarean (*N* = 73)	**EPDS** was positively associated with PBQ-3 (*r* = 0.262). **PBQ Total** was negatively associated with IBM Care (*r* = − 0.443), SWLS-H (*r* = − 0.571), WHO-5 (*r* = − 0.592), RAS-H (*r* = − 0.329), self-efficacy (*r* = − 0.419), MOS-SSS Total (*r* = − 0.378; MOS-1 to MOS-3: *r* = − 0.250 to − 0.474), and positively associated with IBM Control (*r* = 0.319)

The table summarizes statistially significant Pearson correlation coefficients (*p* < 0.05) within delivery mode subgroups For clarity, only correlations with an absolute of *r* > 0.25 are reported. Abbreviations correspond to those defined in Tables 2 and 3. Variables shown in bold represent theoretically central outcome indicators and key constructs of postpartumadjustment, highlighted to support interpretability.

### Psychological and sexual outcomes by postpartum timing and pregnancy risk

Independent-samples t tests were used to examine whether psychological and sexual outcomes differed by time since childbirth, parity, and pregnancy-risk status. For clarity, only statistically significant results (*p* < 0.05) are reported. Time since childbirth was categorized as within the first postpartum year (≤ 12 months) versus the second postpartum year (13–24 months).

### Time since childbirth

Mothers assessed 13–24 months postpartum reported significantly lower scores in several domains of sexual functioning compared to those assessed within the first year postpartum, including lubrication (FSFI; M = 3.27 vs. 3.97; t(158.4) = 2.01, *p* = 0.046), orgasm (M = 2.80 vs. 3.60; t(203) = 2.40, *p* = 0.017), and pain (M = 2.93 vs. 4.08; t(203) = 3.29, *p* = 0.001).

### Parity

Primiparous mothers reported lower depressive symptom scores than multiparous women, both in the full sample (BDI; M = 11.28 vs. 12.69; t(673) = 2.085, *p* = 0.037), and within the first postpartum year (M = 10.72 vs. 12.35; t(428) = 1.964, *p* = 0.05).

### Depression severity subgroups

Among women with mild depressive symptoms (BDI), those assessed beyond the first postpartum year reported higher perceived partner care (t(203) = − 2.765, *p* = 0.006), higher relationship satisfaction t(203) = − 2.006, *p* = 0.046), higher life satisfaction (t(203) = − 2.901, *p* = 0.004), and lower overall sexual functioning (t(203) = 2.143, *p* = 0.033).

Within the moderate depression subgroup, mothers assessed more than one year postpartum reported lower lubrication (t(61.43) = 2.214, *p* = 0.031) and higher pain scores (t(59.26) = 2.723, *p* = 0.008).

In the non-depressed subgroup, women assessed beyond one year postpartum showed higher scores on the PBQ Rejection and anger subscale (t(144.21) = − 2.222, *p* = 0.028). No significant differences were observed across postpartum timing within the severe depression subgroup.

### Pregnancy risk status

Women classified as having high-risk pregnancies reported significantly higher depressive symptom scores than those without pregnany risk (M = 13.10 vs. 11.50; t(673) = − 2.301, *p* = 0.022).

### Psychological correlates of pregnancy complications

To explore the psychological correlates associated with specific obstetric complications, subgroup analyses were conducted among mothers reporting major pregnancy-related health conditions. Only statistically significant associations (*p* < 0.05) are reported.

### Threatened preterm birth

Among mothers with threatened preterm birth (*N* = 21), depressive symptoms were positively correlated with bonding difficulties, particularly caregiving anxiety and perceived risk of abuse (PBQ subscales 3–4; *r* = 0.462–0.503). PTSD symptoms were negatively associated with several domains of sexual functioning, including arousal, lubrication, and orgasm (*r* = − 0.432 to − 0.474).

### Gestational diabetes

In the gestational diabetes subgroup (*N* = 69), depressive symptoms were negatively correlated with self-efficacy, life satisfaction, and perceived social support (*r* = − 0.237 to − 0.304). Higher perceived partner care (IBM-Care) was negatively associated with bonding difficulties across PBQ total and subscale scores (PBQ total and subscales; *r* = − 0.325 to − 0.531).

### Hypertensive disorders

Among women with hypertensive disorders (*N* = 30), partner care (IBM-Care) scores were positively correlated with rejection-related bonding difficulties (PBQ subscale 2; *r* = 0.366). Self-efficacy was negatively associated with overall bonding difficulties and several PBQ subscales (*r* = − 0.359 to − 0.393) and positively associated with psychological well-being (WHO-5; *r* = 0.449).

### Vaginal infections during pregnancy

Among mothers reporting vaginal infections during pregnancy (*N* = 28), depressive symptoms were positively correlated with bonding impairments (PBQ subscale 2; *r* = 0.385). Self-efficacy showed strong negative associations with bonding difficulties (PBQ total and subscales 1–3; *r* = − 0.432 to − 0.719), and with perceived partner control (IBM-Control; *r* = − 0.416).

### History of miscarriage

Among women with a history of miscarriage (*N* = 28), EPDS scores were negatively correlated with sexual lubrication (*r* = − 0.414), pain (*r* = − 0.381), and total FSFI scores (*r* = − 0.375), and positively correlated with bonding difficulties (PBQ total: *r* = 0.372; subscale 1: *r* = 0.403; subscale 2: *r* = 0.374). Perceived partner care (IBMCare) was positively associated with relationship satisfaction (*r* = 0.857), life satisfaction (*r* = 0.507), mental well-being (*r* = 0.601), and sexual satisfaction (*r* = 0.510). Self-efficacy was positively correlated with perceived social support (MOS-SSS total: *r* = 0.524) and negatively with PBQ subscale 4 (*r* = − 0.377).

### Hospitalization during pregnancy

Finally, among mothers who were hospitalized during pregnancy (*N* = 36), both EPDS and BDI scores were positively correlated with bonding disturbances on PBQ subscale 4 (*r* = 0.374 and *r* = 0.410, respectively).

### Regression-based associations with bonding, social support, and relationship satisfaction

Three multiple linear regression models were conducted to examine associations between psychological, relational, and demographic variables and key pospartum outcomes.

### Model 1: maternal bonding (PBQ total)

The model explained 27.9% of the variance in bonding difficulties (R² = 0.279; F(33, 583) = 6.828, *p* < 0.001). Higher self-efficacy (B = − 0.307, *p* < 0.001) and better mental well-being (WHO-5; B = − 0.826, *p* < 0.001) were associated with fewer bonding difficulties. Depressive and PTSD symptoms were not significantpredictors in full model.

### Model 2: perceived social support (MOS-SSS total)

This model accounted for 44.6% of the variance (R² = 0.446; F(31, 585) = 15.174, *p* < 0.001). Greater perceived social support was associated with higher partner care (IBM-Care; B = 0.491, *p* < 0.001), greater life satisfaction (SWLS-H; B = 0.597, *p* < 0.001), and higher mental well-being (WHO-5; B = 1.128, *p* < 0.001).

### Model 3: relationship satisfaction (RAS-H total)

The model explained 79.1% of the variance in relationship satisfaction (R² = 0.791; F(32, 584) = 69.006, *p* < 0.001). Higher relationship satisfaction was associated with greater partner care (IBM-Care; B = 0.424, *p* < 0.001), lower partner control (IBM-Control; B = − 0.142, *p* < 0.001), and greater life satisfaction (SWLS-H; B = 0.216, *p* < 0.001). Among social support dimensions, positive social interaction was positively associated (B = 0.232, *p* < 0.001), whereas emotional/informational (B = − 0.108, *p* < 0.001) and instrumental support (B = − 0.133, *p* = 0.004) were negatively associated with relationship satisfaction (Table 5).

**Table 5:** Linear regression models predicting maternal bonding, perceived social support, and relationship satisfaction.

Outcome variable	Predictor	B	*p*	*R*²	Adjusted *R*²
PBQ Total score				**0.279**	**0.238**
	Self-efficacy	–0.307	< 0.001		
	Mental well-being (WHO-5)	–0.826	< 0.001		
MOS-SSS Total				**0.446**	**0.416**
	Partner care (IBM care)	0.491	< 0.001		
	Life satisfaction (SWLS-H)	0.597	< 0.001		
	Mental well-being (WHO-5)	1.128	< 0.001		
RAS-H Total				**0.791**	**0.779**
	Partner care (IBM care)	0.424	< 0.001		
	Partner control (IBM control)	–0.142	< 0.001		
	Life satisfaction (SWLS-H)	0.216	< 0.001		
	Emotional/informational support	–0.108	< 0.001		
	Positive social interaction	0.232	< 0.001		
	Instrumental support	–0.133	< 0.001		

Note. Results of multiple linear regression analyses predicting postpartum outcomes. All models were statistically significant (*p* < 0.001). B represents unstandardized regression coefficients. R² indicates the proportion of variance explained by the model; Adjusted R² reflects variance explained after adjustment for the number of predictors. *PBQ* Postpartum Bonding Questionnaire, *MOS-SSS* Medical Outcomes Study Social Support Survey, *RAS-H* Relationship Assessment Scale (Hungarian version), *IBM* Intimate Bond Measure, *SWLS-H* Satisfaction with Life Scale (Hungarian version), *WHO-5* WHO-5 Well-Being Index. Values shown in bold indicate overall model fi t indices and are highlighted for clarity and transparency.

## Discussion

This study provides a comprehensive analysis of postpartum psychological and relational challenges, examining the interrelations among depressive symptoms, childbirth-related post-traumatic stress disorder (CB-PTSD), maternal–infant bonding, sexual functioning, and perceived partner and social support. The findings align with previous international research^21,27,29^ and extend existing knowledge by emphasizing the interconnected roles of partner relationship dynamics and maternal self-efficacy as key protective mechanisms shaping postpartum adjustment. Overall, the findings supported both study hypotheses. Lower levels of perceived partner support and maternal self-efficacy were consistently associated with higher depressive symptoms severity and greater bonding difficulties across correlational, multivariate, and regression-based analyses. In addition, childbirth-related PTSD symptoms were linked to poorer sexual functioning, impaired maternal-infant bonding, and reduced perceived partner care, with subgroup analyses indicating contextual variability across relationship duration.

The prevalence of depressive symptoms in our sample (29.6% on the EPDS; 8.9% severe per the BDI) was substantially higher than previous Hungarian estimates (7.1–10.8%)^9,10^ and international averages for high-income countries (13–19%)^6^. This elevation may reflect contextual factors, as data were collected during the COVID-19 pandemic a period marked by isolation, uncertainty, and reduced access to health and social support services, all known to heighten postpartum distress^7^. Pandemic-specific stressors such as fear of infection, restrictions on partner presence during childbirth, and limited professional care may have amplified anxiety and disrupted relational support networks. These circumstances likely contributed to the increased prevalence of depressive symptoms and sexual difficulties observed in our sample. These results therefore likely reflect a contextually heightened level of distress rather than normative population rates. Future studies are needed to assess whether similar patterns persist under typical postpartum conditions.

Although prior research suggests that postpartum depression (PPD) tends to be less prevalent in high-income settings^2,6^, our findings indicate that favorable socioeconomic conditions alone do not safeguard against psychological risk. Instead, individual psychosocial resources—particularly partner support, maternal self-efficacy, and relationship quality—appear to be central in determining postpartum adaptation. When these supports are limited or misaligned, mothers may remain vulnerable to distress, even within supportive healthcare systems. This underscores that relational and emotional safety, rather than material resources, are the most immediate buffers against postpartum depression.

The observed rate of CB-PTSD (4.6%) was consistent with previous international estimates (0–7%)^3030,3232^, and its strong correlation with depressive symptoms (*r* = 0.665) confirms their well-documented comorbidity^27^. CB-PTSD symptoms were linked to impaired bonding and reduced partner intimacy, suggesting that traumatic birth experiences can disrupt both emotional attunement and relational closeness. Mothers who re-experience intrusive memories or heightened physiological arousal may struggle with physical or emotional intimacy, which can indirectly affect maternal sensitivity and bonding.

Bonding difficulties were associated with depressive symptoms, lower self-efficacy, and reduced well-being and social support. Higher „Impaired bonding” and „Rejection and anger” scores were particularly evident among mothers with low self-efficacy or elevated PTSD symptoms, consistent with prior findings on the emotional and relational determinants of bonding^24,27^. After controlling for obstetric and demographic factors, depressive symptoms—but not PTSD—remained a significant independent predictor of bonding difficulties. This suggests that the association between childbirth-related trauma and bonding may be largely accounted for by concurrent depressive processes or obstetric factors. Notably, the EPDS, designed for perinatal use, proved more sensitive to these patterns than the general BDI, supporting the value of using perinatal-specific screening tools. Importantly, higher partner and social support, greater relationship satisfaction, and better sexual functioning were independently associated with fewer bonding problems, underscoring the protective value of supportive couple relationships and positive relational engagement.

Maternal self-efficacy showed a strong, consistent negative association with bonding difficulties across all PBQ domains and emerged as a robust correlate of adaptive functioning. Higher self-efficacy was associated with greater emotional stability and perceived competence, while lower self-efficacy corresponded to uncertainty and reduced maternal confidence. Partner relationship quality also played a central role: higher perceived partner care predicted well-being, life satisfaction, and bonding security, whereas controlling behaviors were linked to distress, bonding impairment, and sexual dissatisfaction^50,56,57^. These findings highlight how supportive partner behavior may foster maternal confidence and emotional regulation, while critical or controlling dynamics can undermine autonomy and perceived competence. Thus, postpartum adjustment is best conceptualized as a relational process shaped by mutual responsiveness and emotional availability.

Sexual dysfunction affected 32.1% of participants and showed clear associations with depressive and PTSD symptoms. Lower lubrication, orgasm, and satisfaction scores were most evident during the second postpartum year, suggesting that sexual difficulties may persist or intensify over time. Consistent with earlier findings^21,44^, sexual functioning was not only related to mood but also to relational and body-related experiences. Importantly, sexual functioning was linked to maternal-infant bonding—particularly in the “Rejection and anger” and “Risk of abuse” domains—implying that body-related distress and emotional withdrawal may interfere with caregiving sensitivity and intimacy. These findings expand understanding of the interdependence between relational and maternal functioning in the postpartum period.

The strong correlation between relationship satisfaction and perceived partner care (r = 0.851) emphasizes the protective value of empathy and emotional support within partnerships. Partner care predicted higher self-efficacy, life satisfaction, and bonding security, whereas partner control was inversely related to these outcomes. Regression analyses identified partner care, mental well-being, and life satisfaction as the strongest predictors of perceived social support and relationship quality. Interestingly, emotional/informational and instrumental support were negatively associated with relationship satisfaction. This paradox likely reflects situations in which received support does not match the mother’s needs or occurs in compensatory contexts where partner responsiveness is low. Excessive or poorly timed assistance may, despite good intentions, inadvertently undermine maternal autonomy or sense of competence. Among the subdimensions of social support, „Positive Social Interaction” was most consistently linked with maternal well-being and relationship satisfaction, highlighting that shared daily experiences and emotional connection may offer the most meaningful form of support^4,46^.

Beyond the primary findings, the subgroup analyses revealed several contextual factors influencing postpartum adaptation. Mothers with medically classified high-risk pregnancies reported higher depressive symptoms^39^, though stable partner support appeared to buffer these effects^37,38^. Women in mid-length relationships (4–10 years) reported lower self-efficacy, poorer sexual functioning, and higher PTSD symptoms than those in longer-term partnerships, suggesting that relationship maturity and established emotional communication may protect against postpartum distress^33,40^. Emergency caesarean birth was associated with caregiving anxiety and bonding difficulties, particularly among mothers with lower self-efficacy or limited support, reinforcing the need for trauma-informed postpartum care. Together, these findings underscore that maternal mental health is embedded in a dynamic interplay of psychological, relational, and obstetric factors rather than isolated individual processes. Comprehensive postpartum care that integrates relational, emotional, and medical dimensions is therefore essential to promote maternal well-being and secure bonding.

### Limitations and strengths

This study provides valuable insight into the psychosocial aspects of the postpartum period; however, several methodological limitations should be considered. First, the cross-sectional design precludes causal inference and limits conclusions regarding the directionality of the observed associations among postpartum depression, childbirth-related PTSD (CB-PTSD), bonding difficulties, sexual functioning, and perceived social support. Longitudinal studies are needed to clarify temporal and bidirectional relationships between maternal mental health, bonding, and relationship quality.

Second, reliance on self-report questionnaires may have introduced response bias due to social desirability or stigma, particularly regarding depressive symptoms, sexual functioning, and relationship satisfaction. Future research could incorporate clinician-rated or qualitative data to enhance validity.

Third, the sample was not fully representative of the Hungarian postpartum population. Participants were predominantly urban, highly educated, and in stable relationships, limiting generalizability to more diverse or vulnerable groups such as single mothers, those of lower socioeconomic status, or women in rural regions with limited healthcare access. These populations may face additional barriers to support and higher psychosocial risk.

Data collection during the COVID-19 pandemic may also have influenced results. Pandemic-related restrictions, social isolation, and limited partner presence during childbirth likely contributed to elevated psychological distress and sexual dysfunction. Consequently, the prevalence rates may reflect the exceptional context of the pandemic rather than typical postpartum conditions.

Although the inclusion of partner relationship dynamics and maternal self-efficacy represents a strength, these constructs were measured solely from the maternal perspective, and at a single time point. Without dyadic or longitudinal data, the reciprocal nature of these processes remains only partially understood. Future studies should adopt multi-informant and couple-based designs to better capture relational influences on maternal adjustment.

Despite these limitations, this study has notable strengths. It represents the first large-scale Hungarian investigation (*N* = 675) to comprehensively examine postpartum depression, CB-PTSD, maternal bonding, sexual functioning, and perceived partner and social support within an integrated psychosocial framework. The use of validated multidimensional measures and adjusted regression models allowed for a nuanced assessment of both risk and protective factors, including maternal self-efficacy and partner care, which emerged as key psychological protective factors.

Moreover, the study revealed novel associations that warrant further exploration, such as the links between sexual functioning and maternal bonding, and the complex patterns between emotional or instrumental support and relationship satisfaction. These findings open new directions for understanding how interpersonal and psychological factors jointly influence postpartum adaptation.

Finally, although Hungary is a high-income country, the high prevalence of depressive symptoms (29.6%) and sexual dysfunction (32.1%) highlights that socioeconomic stability alone does not ensure maternal well-being. Together, these findings underscore the need for integrated, trauma-informed, and relationship-focused postpartum care addressing both individual and interpersonal dimensions of maternal health.

### Implications for practice

Our findings underscore that postpartum mental health and mother–infant bonding are embedded in a complex psychosocial system where partner support, maternal self-efficacy, and relationship quality play central roles. Therefore, postpartum screening may benefit from extending beyond the assessment of depressive symptoms to include relational aspects such as couple dynamics, sexual functioning, and perceived maternal competence. Integrating EPDS-based screening into routine postpartum care could facilitate early identification and targeted psychosocial support for at-risk mothers.

Higher maternal self-efficacy was consistently associated with lower levels of depression, bonding difficulties, and perceived social isolation, suggesting that interventions aimed at strengthening maternal confidence and competence—through psychoeducation, group-based programs, or individualized support—may serve as potentially effective preventive strategies to reduce postpartum distress. Similarly, enhancing perceived partner care and relational security may function as key protective factors for maternal well-being. The observed negative associations between emotional/instrumental support and relationship satisfaction indicate that support quality, timing, and fit may be more critical than quantity alone. This highlights the need for a paradigm shift in postpartum support systems, emphasizing the contextual sensitivity and appropriateness of provided help.

It is equally important to address barriers to help-seeking. Many mothers hesitate to access professional care due to feelings of inadequacy, guilt, or the normalization of distress as a „natural” part of motherhood. Reducing stigma, increasing public awareness, and promoting mental health literacy could facilitate earlier recognition and engagement with support services.

In light of these findings, psychosocial interventions may be particularly beneficial when they adopt a dyadic focus, involving both partners, particularly in cases of sexual dissatisfaction, relationship strain, or unresolved birth-related trauma. Couple-based therapy, sexological counseling, and trauma-informed psychological care may alleviate sexual dysfunction, strengthen bonding, and enhance relationship quality. Such approaches are particularly relevant for high-risk groups, including women with medically complex pregnancies, emergency caesarean deliveries, or those transitioning to motherhood for the first time.

Given the complexity of postpartum adaptation, an interdisciplinary care model is essential. Collaboration among obstetric, midwifery, nursing, psychological, and social work professionals can facilitate early detection and comprehensive support for mothers at risk of depression, bonding difficulties, or relational distress. Strengthening mother–infant bonding thus requires parallel attention to maternal psychosocial resources and the relational context within which early caregiving unfolds.

## Conclusion

This study demonstrates that postpartum depression—particularly when measured with the EPDS—emerged as a stronger psychological correlate of impaired maternal bonding than childbirth-related PTSD symptoms in adjusted analyses. In contrast, partner support, relationship satisfaction, and sexual functioning emerged as independent protective factors, with sexual functioning also showing significant associations with maternal–infant bonding quality. As data collection occurred during the COVID-19 pandemic, the observed prevalence of distress and sexual difficulties should be interpreted cautiously, as these rates may not generalize beyond pandemic conditions. Clinically, integrating EPDS-based screening with brief assessments of relational and sexual domains could improve early detection and tailored support for at-risk mothers. Strengthening maternal self-efficacy and partner relationships appears crucial for promoting both maternal well-being and healthy mother–infant bonding.

## Data Availability

The datasets generated and/or analysed during the current study are available from the corresponding author on reasonable request.
